# Hypothermia-Related Deaths — Wisconsin, 2014, and United States, 2003–2013

**Published:** 2015-02-20

**Authors:** Jon Meiman, Henry Anderson, Carrie Tomasallo

**Affiliations:** 1 Epidemic Intelligence Service, CDC; 2 Bureau of Environmental and Occupational Health, Wisconsin Division of Public Health

Hypothermia is defined as a core body temperature of <95°F (<35°C) and is caused by environmental exposure, drug intoxication, or metabolic or nervous system dysfunction. Exposure to cold is a leading cause of weather-related mortality and is responsible for approximately twice the number of deaths annually as exposure to heat in the United States (1). To understand the risk factors for hypothermia-related death and improve prevention efforts, during January 1–April 30, 2014, a period of record low temperatures, the Wisconsin Division of Public Health began active surveillance for hypothermia. Suspected hypothermia-related deaths were reported by coroners or medical examiners and identified in death records. Hypothermia was confirmed as the cause of death by review of death investigation narratives. This report describes three selected cases of hypothermia-related deaths in Wisconsin and summarizes characteristics of all cases that occurred in the state during the period of active surveillance. A summary of hypothermia-related deaths for the United States during 2003–2013 also is presented for comparison and to assess national mortality trends. Hypothermia continues to be an important cause of weather-related death. Key risk factors include drug intoxication, mental illness, and social isolation. State and local health agencies might need to focus outreach on vulnerable populations and target interventions for groups at highest risk for death.

## Case Reports

### Case 1

In January 2014, a man aged 25 years was found frozen to death one block from his home. The decedent had been healthy previously and had no known medical conditions. Ambient temperature at the estimated time of death was −8°F (−22°C). Death investigation concluded that he had been dropped off at the wrong residence after leaving a tavern. His blood alcohol level was 230 mg/dL (intoxication is legally defined as ≥80 mg/dL in all states), and cause of death was environmental hypothermia with a contributing cause of alcohol intoxication.

### Case 2

In February 2014, a deceased woman aged 59 years was found outside in her driveway 72 hours after last contact with a friend. She lived alone and had multiple comorbid conditions, including type 2 diabetes, chronic obstructive pulmonary disease, and spinal stenosis. Ambient temperature at the estimated time of death was 6°F (−14°C). The death investigation concluded that she likely fell and sustained minor injuries. Although she was wearing suitable clothing for the weather, she was unable to stand because of impaired mobility. The cause of death was environmental hypothermia.

### Case 3

In March 2014, a deceased man aged 63 years was found in a snow-covered field. The decedent had a history of advanced Parkinson’s disease and lived alone. Family members reported that he had been unable to care for himself completely, and neighbors noted he had a tendency to wander outdoors. He had last spoken with his family 36 hours before discovery of his body. Core body temperature was 42°F (6°C), and the ambient temperature at time of discovery was 35°F (2°C). The decedent was wearing only jeans, a short-sleeve shirt, shoes, and gloves. Autopsy and death investigation concluded the cause of death was environmental hypothermia.

## Wisconsin, 2014

During January–April 2014, a total of 27 hypothermia-related deaths occurred in Wisconsin, all of which were investigated by a coroner or medical examiner. Eighteen (67%) decedents were male; decedents’ median age was 66 years (range = 25–95 years). Autopsies were performed on 14 (52%) decedents, and toxicology was performed for nine (33%). Of those nine, six (67%) were positive for alcohol, and one (11%) was positive for both a prescription opioid and delta-9-tetrahydrocannabinol (the principal psychoactive ingredient of cannabis). Eighteen (67%) bodies were discovered outdoors, and the median outside temperature at the estimated time of death was 6°F (−15°C), with a range of −14°F to 35°F (−26°C to 2°C). Four (15%) of the decedents who were discovered indoors resided in homes with unused or nonfunctional furnaces. Coroner and medical examiner investigations revealed that five (19%) decedents had a history of mental illness. Fifteen (56%) lived alone, and two (7%) had been homeless.

## United States, 2003–2013

Hypothermia-related deaths for the United States overall were obtained from CDC’s multiple cause of death files and were defined as any death with an underlying or contributing cause of death from exposure to excessive natural cold (*International Classification of Diseases*, *10th Revision* [ICD-10] code X.31). A total of 13,419 deaths occurred during the period, with unadjusted annual rates ranging from 0.3 to 0.5 per 100,000 persons. There was a statistically significant increase in rates over the period (chi-square for trend; p<0.01). Males accounted for 9,050 (67%) decedents. Rates of death were highest among persons of advanced age; mean death rates during the 10-year period for males and females aged ≥65 years were 1.8 and 1.1 per 100,000 population, respectively (Figure). A total of 1,391 (10%) decedents had alcohol or drug poisoning (ICD-10 codes X.40–45, Y.10–15, or F.10) as a contributing cause of death.

**What is already known on this topic?**

Exposure to extreme cold is a leading cause of preventable weather-related mortality in the United States. Risks for hypothermia-related death include advanced age, mental illness, male sex, and drug intoxication.

**What is added by this report?**

During January–April 2014, a total of 27 hypothermia-related deaths occurred in Wisconsin. Eighteen decedents were male; median age was 66 years (range = 25–95 years). Six of nine tested for alcohol were positive. Eighteen bodies were discovered outdoors. Investigations revealed that five decedents had a history of mental illness, 15 lived alone, and two had been homeless. Rates of hypothermia-related deaths in the United States increased during 2003–2012.

**What are the implications for public health practice?**

Social isolation can be an important factor in hypothermia-related mortality. State and local health agencies might need to target public education and interventions at socially isolated groups, including older persons, the homeless, and those living alone.

### Discussion

Hypothermia is a preventable cause of death that begins when core body temperature decreases to <95°F (<35°C). Initial symptoms include shivering and cool extremities. As hypothermia worsens, symptoms progress to confusion, loss of fine motor skills, and amnesia (2). Continued heat loss without adequate rewarming can result in hypotension, impaired respiration, cardiac arrhythmias, and death (2).

This report highlights previously identified risk factors for fatal hypothermia, including advanced age, male sex, drug intoxication, homelessness, and mental illness. Older persons have impaired heat generation and often multiple comorbidities that increase the risk for death (3). Substance and alcohol abuse can contribute to hypothermia by blunting physiologic responses to cold and can lead to prolonged exposure caused by impaired judgment. A review of Wisconsin hypothermia-related deaths revealed that approximately half of decedents lived alone, which can lead to substantial delays in treatment if persons are incapacitated by injury or illness.

Prompt recognition of the signs and symptoms of hypothermia is necessary for reducing mortality. Patients should be rewarmed by using external warming (e.g., blankets or forced heated air) for mild hypothermia and internal warming methods (e.g., body cavity lavage) for severe hypothermia. In the event of cardiac arrest, cardiopulmonary resuscitation should be performed during rewarming in accordance with published guidelines (4).

Greater awareness of severe weather events and the need for emergency and disaster response has led to increased public health attention to weather-related response planning (5). One of the most visible components of such plans is the opening of publically available warming shelters when extreme cold is expected. However, this report suggests that state and local health agencies also might need to focus more on public education, communication networks to reach the most vulnerable persons, and targeted interventions for groups at risk (e.g., older persons, homeless, and those living alone). Educational materials should emphasize the rapidity with which hypothermia can occur, review the warning signs of hypothermia, and outline ways to reduce risk (e.g., wearing suitable clothing, avoiding hazardous weather situations, and preparing for such emergencies as motor vehicle breakdowns and power outages). Emphasizing how alcohol consumption and certain drugs increase the risk for cold-related injuries and hypothermia also might be helpful.

## Figures and Tables

**FIGURE:**
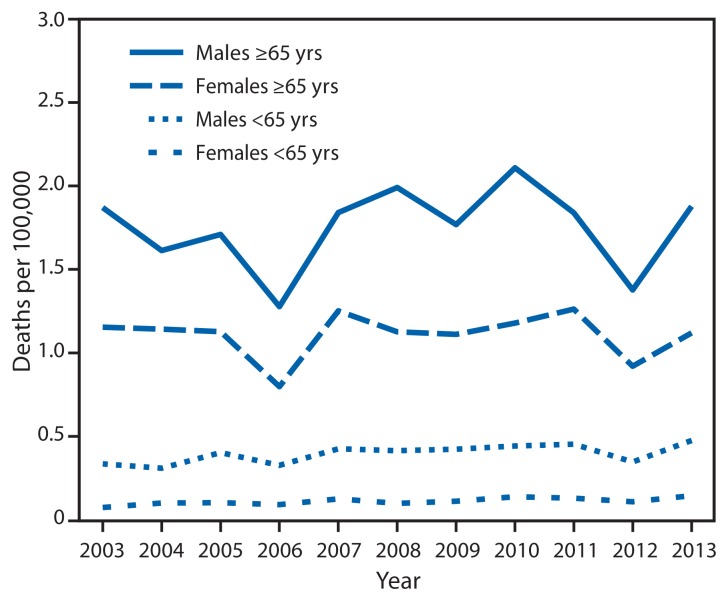
Rate* of hypothermia-related deaths,^†^ by sex and age group — United States, 2003–2013 * Deaths per 100,000 persons. ^†^ Hypothermia-related deaths were defined as any with an underlying or contributing cause of death from exposure to excessive natural cold (*International Classification of Diseases, 10th Revision* code X.31).

## References

[1] Berko J, Ingram DD, Saha S (2014). Deaths attributed to heat, cold, and other weather events in the United States, 2006–2010. Natl Health Stat Report.

[2] Aslam AF, Aslam AK, Vasavada BC, Khan IA (2006). Hypothermia: evaluation, electrocardiographic manifestations, and management. Am J Med.

[3] Jurkovich GJ (2007). Environmental cold-induced injury. Surg Clin North Am.

[4] Vanden Hoek TL, Morrison LJ, Shuster M (2010). Part 12. Cardiac arrest in special situations: 2010 American Heart Association guidelines for cardiopulmonary resuscitation and emergency cardiovascular care. Circulation.

[5] Marinucci G, Luber G, Uejio C, Saha S, Hess J (2014). Building resilience against climate effects—a novel framework to facilitate climate readiness in public health agencies. Int J Environ Res Public Health.

